# Cholesterol and Its Oxidation Derivatives Content in Market Dairy Products

**DOI:** 10.3390/nu16091371

**Published:** 2024-04-30

**Authors:** Małgorzata Czerwonka, Anna Gielecińska, Agnieszka Białek, Małgorzata Białek, Barbara Bobrowska-Korczak

**Affiliations:** 1 School of Health and Medical Sciences, University of Economics and Human Sciences in Warsaw, Okopowa 59, 01-043 Warsaw, Poland; a.bialek@vizja.pl; 2 Department of Toxicology and Food Science, Faculty of Pharmacy, Medical University of Warsaw, Banacha 1, 02-097 Warsaw, Polandbarbara.bobrowska@wum.edu.pl (B.B.-K.); 3 The Kielanowski Institute of Animal Physiology and Nutrition, Polish Academy of Sciences, Instytucka 3, 05-110 Jabłonna, Poland; m.bialek@ifzz.pl

**Keywords:** cholesterol, cholesterol oxidation derivatives, dairy products, cheese, yoghurt

## Abstract

Cholesterol oxidation products (COPs) are contaminants of food of animal origin. Increased levels of these compounds in the human body are associated with an increased risk of many non-communicable diseases. Dairy products are mentioned among the main sources of these compounds in the diet. The objective of this study was to evaluate the contents of cholesterol and its oxidized derivatives in eleven groups of dairy products, willingly consumed in European countries. The levels of COPs were determined by applying the GC-TOF/MS method. In the tested products, cholesterol and its oxidation derivatives, such as 7-ketocholesterol, 7α-hydroxycholesterol, 7β-hydroxycholesterol, 5,6β-epoxycholesterol and 5,6α-epoxycholesterol, were determined. The studied dairy products differed in their contents and profiles of oxysterols. The highest contents of COPs were found in cheese with internal mold (13.8 ± 2.5 mg kg^−1^) and Cheddar (11.7 ± 3.5 mg kg^−1^), while the lowest levels were detected in yoghurt (0.94 ± 0.30 mg kg^−1^) and kefir (0.57 ± 0.11 mg kg^−1^). 7-ketocholesterol and 5,6β-epoxycholesterol were the dominant oxysterols. The ratio of oxidized derivatives to total cholesterol was on average 1.7%. Our results confirmed that dairy products are an important dietary source of COPs. Their levels should be monitored in dairy products to provide the best health quality.

## 1. Introduction

Cholesterol is a steroid compound characteristic of food of animal origin. For years, scientific works have focused mostly on the link between its content in the diet and the occurrence of many non-communicable diseases [1]. However, it has been established that approximately 20% of total cholesterol in humans is secured from the diet and 80% is normally biosynthesized in the body [2]. Cholesterol has many essential physiological functions, e.g., bile acid synthesis, the precursor of vitamin D and steroid hormones synthesis, sperm development, functioning of the immune system and central nervous system, maintaining the structural integrity of cells, and impact on membrane fluidity and trans-membrane signaling pathways [3]. Nevertheless, lately, the research focus has shifted from the cholesterol itself to the products of its oxidation (COPs) [4]. Cholesterol easily undergoes oxidation, both enzymatically and non-enzymatically driven, which results in oxysterols’ formation, differing from the parent molecule for an epoxy, hydroxyl or ketone group in the steroid nucleus or a hydroxy group in the hydrophobic side chain. In the human body, the activity of numerous enzymes, usually mitochondrial or microsomal enzymes belonging to the cytochrome P450 family, leads 27-hydroxycholesterol, 24-hydroxycholesterol, 7α-hydroxycholesterol, 20α-hydroxycholesterol and 25-hydroxycholesterol to arise [5,6]. In the human body, COPs fulfil many important functions. They are intermediates in the synthesis of bile acids, steroid hormones and 1,25-dihydroxycholecalciferol. These compounds also affect cholesterol formation and metabolism, as well as carbohydrate metabolism [7]. However, when levels of COPs in the body are too high, this can have serious detrimental health implications [8]. COPs have proinflammatory and proapoptotic activities, and the role of cholesterol oxidized derivatives in the pathogenesis of cardiovascular, neurodegenerative and other non-communicable diseases has been confirmed [9,10].

Oxysterols in organisms may be formed directly in the body not only as a result of enzymatic cholesterol transformations but also due to the activity of reactive oxygen species (ROS), whose elevated levels accompany different pathological conditions and diseases, usually of inflammatory origin [11]. Non-enzymatic oxidation of cholesterol is also induced in food products by factors related to their manufacturing and processing such as heat, light exposure, maturation, refrigeration, freeze-drying and spray-drying [12]. It leads to formation of different COPs: 7β-hydroxycholesterol, 7-ketocholesterol, 5α,6α-epoxycholesterol, 5β,6β-epoxycholesterol and cholestan-3β,5α,6β-triol [6]. COPs from the diet are better absorbed in the digestive tract than cholesterol and can increase the total pool of COPs in the body [13]. Excessive levels of COPs have been associated with involvement in chronic inflammatory processes [9]. Both in vitro and animal studies have shown that dietary COPs may play a role in the pathogenesis of diseases such as cardiovascular disease (atherosclerosis, hypertension), Parkinson’s disease, cancers (breast, ovarian, lung, colon), cataracts, diabetes and diabetic nephropathy, osteoporosis and many others [14].

Milk and dairy products have long been known as rich dietary sources of numerous nutrients, including proteins and peptides, lipids (conjugated fatty acids, natural *trans* fatty acids and odd- and branch-chained fatty acids), minerals (Ca, Cu, Fe, K, Mg, Mn, Na, P, Se and Zn), vitamins (A, B_1_, B_2_, B_3_, B_6_, B_9_, B_12_, D, E and K) as well as probiotic microorganisms with a beneficial influence on health [15]. Nowadays, the consumption of low-fat dairy products is advocated in most dietary guidelines around the world, as opposed to full-fat products. Dairy lipids are rich in saturated fatty acids and therefore have the potential to increase the risk of cardiovascular disease [16]. Moreover, dairy products, due to their high intake, may be an important source of COPs in the diet, as fermentation and/or ripening processes, sometimes lasting for several years, can promote the oxidation of lipid compounds, including cholesterol [17]. Studies referring to the contents of COPs in milk and fermented milk products are scarce [18,19], as are studies on the health influences of the dietary intake of COPs from milk and dairy products.

Taking into account those premises, the objective of the present study was to evaluate the contents of cholesterol and its oxidized derivatives in dairy products available on the market.

## 2. Materials and Methods

### 2.1. Research Material

The research material comprised eleven groups of dairy products derived from cow’s milk only, listed in Table 1. In each group, there were 12 products of different brands. The research material came from all over the European Union and was purchased in grocery shops in Warsaw (Poland). Samples were stored under refrigerated conditions and examined before the expiration date. None of the products contained flavor additives. Only products with a regular fat content were under investigation.

### 2.2. Analytical Methods

#### 2.2.1. Reagents

Cholesterol and COP analytical standards, 5α-cholestane (HPLC grade), BSTFA + 1% TMCS (*N*,*O*-Bis(trimethylsilyl)trifluoroacetamide with 1% of trimethylchlorosilane, for GC derivatization) and pyridine (HPLC grade) were purchased from Sigma Aldrich Corp., Poznan, Poland. Methanol (HPLC grade), ethanol (96%, analytical grade), hexane (HPLC grade), chloroform (analytical grade), potassium hydroxide (analytical grade) and BHT (butylated hydroxytoluene, analytical grade) were bought in Avantor Performance Materials Poland S.A., Gliwice, Poland. Nitrogen (purity: ≥99.999%) was provided by Multax S.C., Stare Babice, Poland. Helium (purity: ≥99.9999%) was bought from Air Products and Chemicals, Inc., Warsaw, Poland.

#### 2.2.2. Determination of Cholesterol and Its Oxidized Derivatives

To 200–300 mg of cheese sample (or 50 mg of fat extracted from yoghurt/kefir), 10–15 μL of BHT solution (5 mg mL^−1^ in ethanol) and 3 mL of 1 M KOH in ethanol and 25 μL of internal standard solution (5α-cholestane, 0.5 mg mL^−1^ in hexane) were added and homogenised. Hydrolysis lasted for 20–22 h. After this time, 4 mL of water and 2 mL of hexane were added, and samples were vigorously shaken. The hexane layer was collected into 2 mL vials and evaporated under a stream of nitrogen. Then, 50 µL of pyridine and 25 µL of BSTFA + 1% TMCS were added to the vials and mixed thoroughly. The derivatization process was carried out for 45 min at 80 °C. Then, 225 µL of hexane was added to the sample to isolate the cholesterol and COP trimethylsilyl derivatives to be injected into the column.

Chromatographic separation and detection were carried out using GC-TOF/MS (Pegasus^®^ BT, LECO Corporation, St. Joseph, MI, USA). 1 μL was injected into the column in a splitless mode. The injector temperature was 290 °C. An Rxi^®^-17SilMS (30 m × 0.25 mm × 0.25 μm, Restek, Bellefonte, PA, USA) column was used. The temperature program was as follows: 200 °C—4.60 min; increase 5 °C per min to 290 °C; 290 °C—12.4 min. Helium was used as a carrier gas (flow: 1 mL min^−1^). The transfer line temperature was 290 °C. EI ionization was used (temperature: 250 °C, energy: 70 eV).

Compounds were identified based on retention times and mass spectra of cholesterol and COP trimethylsilyl derivatives, while quantitative analysis was based on standard curves produced for each compound and internal standard. The method has been fully validated; results are shown in our previous publication [20].

#### 2.2.3. Fat Content Determination

The fat contents in samples of dairy products were determined gravimetrically after extraction with a mixture of chloroform/methanol (*v*/*v* 2:1) and solvents’ evaporation under a stream of nitrogen, according to the procedure described by Folch and co-workers [21].

### 2.3. Statistical Analysis

Fat, cholesterol and COP content data are presented as mean values ± standard deviation. Differences in tested compounds’ concentrations among groups and differences in individual COP levels in product groups were assessed using a one-way analysis of variance, followed by Tukey’s honestly significant difference post hoc test. The acceptable level of significance was established at α = 0.05. The relationship between fat, cholesterol and COP content was examined using the Pearson correlation coefficient (r). Cluster analysis of oxysterols’ amounts and profile was also performed. We used the Ward agglomeration procedure as a grouping method and Euclidean distance as a function of the distance. The more restrictive Sneath’s criterion (33%) was used for dendrogram analysis and cluster distinguishing. Statistical analysis was performed using the Statistica 13.3 program (StatSoft, Kraków, Poland).

## 3. Results

Fat, cholesterol and its oxidation products were found in all dairy products. Their contents are shown in Table 2.

The level of lipid fraction in the tested products varied within quite a wide range, depending on the type of product. The highest fat content was detected in English-type cheese (33.5 ± 1.2%), while the lowest were in kefir (1.8 ± 0.3%) and yoghurt (3.5 ± 1.8%). The fat content in English-type cheese significantly exceeded the fat contents in almost all other examined products, except cheeses with internal mold (IM). The cholesterol content in the examined dairy products depended upon the fat content (r = 0.926) and differed between the groups of dairy products. Cholesterol levels varied widely from 38.4 ± 1.9 mg kg^−1^ (K) to over 600 mg kg^−1^ (623 ± 75 mg kg^−1^ in I, 625 ± 61 mg kg^−1^ in E and 639 ± 99 mg kg^−1^ in IM, respectively).

**Figure 1:**
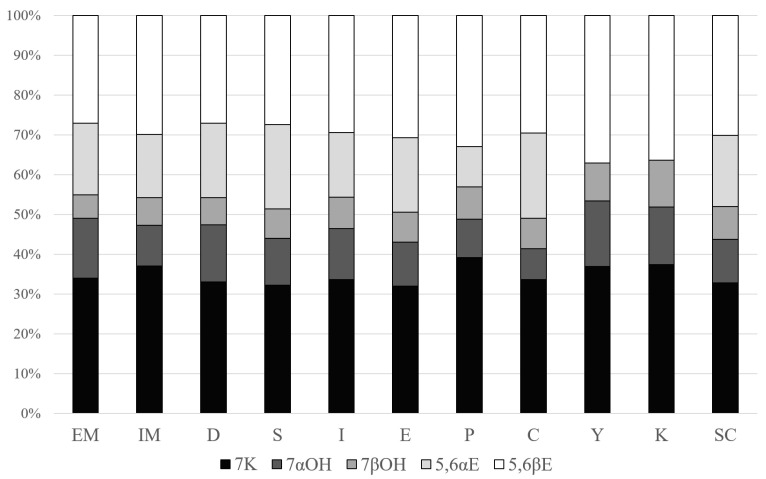
Cholesterol oxidation product profile in dairy products. 7K—7-ketocholesterol; 7αOH—7α-hydroxycholesterol; 7βOH—7β-hydroxycholesterol; 5,6βE—5,6β-epoxycholesterol; 5,6αE—5,6α-epoxycholesterol; EM—cheeses with exterior mold; IM—cheeses with internal mold; D—Dutch-type cheeses; S—Swiss-type cheeses; I—Italian-type cheeses; E—English-type cheeses; P—processed cheeses; C—curd cheeses; Y—yoghurts; K—kefirs; SC—sour cream.

Five oxidized cholesterol derivatives were found in the dairy products: 7-ketocholesterol (7K), 7α-hydroxycholesterol (7αOH), 7β-hydroxycholesterol (7βOH), 5,6β-epoxycholesterol (5,6βE) and 5,6α-epoxycholesterol (5,6αE). All the above-mentioned COPs were identified in most of examined products; however, in some samples, 5,6αE and/or 7αOH derivatives were not present. 5,6αE was not detected at all in samples of yoghurt (Y) and kefir (K) and some single samples of EM, I, P and SC, whereas 7αOH was not noted in single samples of IM, D, S, I, E, P, C, K and SC. The studied product groups differed significantly (*p* < 0.01) in the contents and profile of COPs (Figure 1).

The main oxysterol in dairy products was 7K. In the tested samples, it constituted from 32 ± 3.8% (E) to 40 ± 6.8% (P) of all COPs. Its content ranged from 0.21 ± 0.04 mg kg^−1^ (K) to 3.7 ± 1.2 (E) mg kg^−1^ of the product. The amounts of 7K determined in E, EM and S significantly (*p* < 0.05) exceeded its contents in the other examined dairy products.

Significant amounts of 5,6βE were also detected in products of interest, with this compound ranging from 0.21 ± 0.07 (K) to 4.15 ± 0.91 mg kg^−1^ of the product (IM), which equaled from 27% (EM, D) to 37% (Y) of the total COP content. The contents of this COP in IM and E significantly (*p* < 0.05) exceeded its contents in the other examined products, whereas in the case of Y and K, we found significantly (*p* < 0.05) smaller contents of 5,6βE than the other investigated groups of products.

The contents of 7αOH in dairy products ranged from 0.08 ± 0.03 (K) to 1.4 ± 1.2 mg kg^−1^ (IM) and 1.35 ± 0.16 mg kg^−1^ (EM). The cheeses with internal and external mold were significantly (*p* < 0.05) richer sources of 7αOH than C, Y, K and SC. For Y and K, the amounts of 7αOH were significantly lower than in almost all other examined dairy products, except for C, P and SC. The share of this oxysterol in the total amount of these compounds accounted for 8–16%.

The content of the anomer 7βOH varied at between 0.07 ± 0.03 (K) and 0.97 ± 0.22 mg kg^−1^ (IM), representing 6–12% of total COPs. Similar to the case of the other examined anomer, the amounts of 7βOH determined in EM and IM significantly (*p* < 0.05) exceeded the contents of this COP in the other examined products. Y and K were characterized by much smaller (*p* < 0.05) contents of this COP than the other dairy products of interest.

5,6αE was not identified in the yoghurt and kefir group. In the other products, the contents of this compound ranged from 0.84 ± 0.04 mg kg^−1^ (SC) to 2.19 ± 0.63 mg kg^−1^ (IM), and stood at 2.18 ± 0.41 mg kg^−1^ in E. These products were characterized by a significantly higher content of 5,6αE than in D, I P, C and SC. In the case of SC, the content of 5,6αE was much smaller (*p* < 0.05) than in E, S, IM and EM. The share in the total COP pool for 5,6αE was 10–21%.

**Figure 2:**
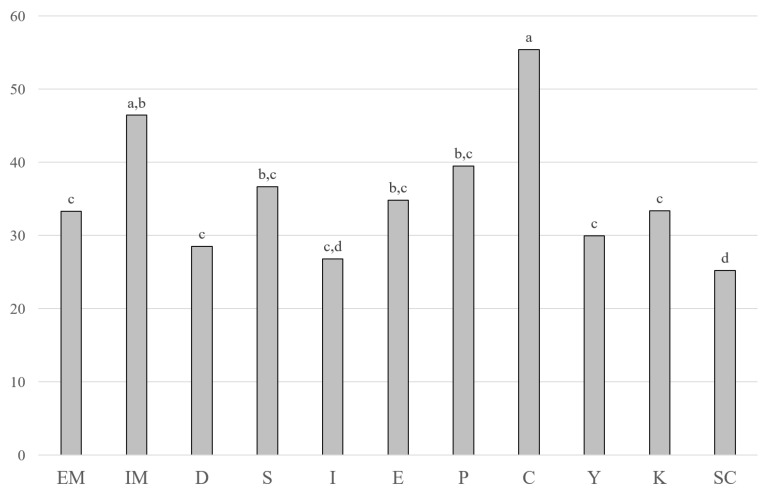
Total cholesterol oxidation product contents in dairy products (mg kg^−1^ of fat). EM—cheeses with exterior mold; IM—cheeses with internal mold; D—Dutch-type cheeses; S—Swiss-type cheeses; I—Italian-type cheeses; E—English-type cheeses; P—processed cheeses; C—curd cheeses; Y—yoghurts; K—kefirs; SC—sour cream. ^a–d^ homogeneous groups; comparison among product groups (Tukey’s test, α = 0.05).

The proportion of oxidized cholesterol derivative content to the parent molecule content in dairy products ranged from 1.3% to 2.3%. The total COP content in the tested groups depended significantly on the total lipid content (r = 0.810) and cholesterol level (r = 0.807; Table 3). However, after calculating COPs in terms of the contents of these compounds in the lipid fraction (Figure 2), significant differences were still observed between the groups of tested products (*p* < 0.05).

**Figure 3:**
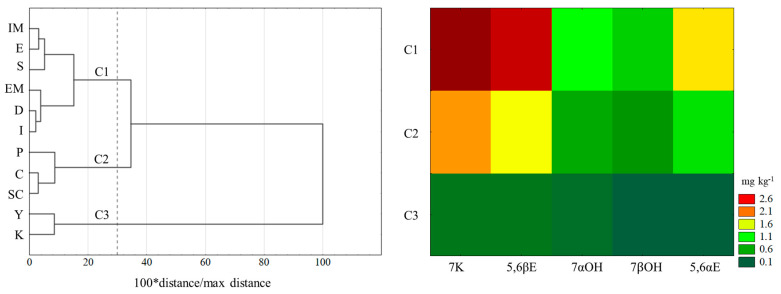
Dendrograms of cholesterol oxidation products (COP) contents in selected dairy products and heatmap of COP levels (mg kg^−1^) in extracted clusters (C1–C3). EM—cheeses with exterior mold; IM—cheeses with internal mold; D—Dutch-type cheeses; S—Swiss-type cheeses; I—Italian-type cheeses; E—English-type cheeses; P—processed cheeses; C—curd cheeses; Y—yoghurts; K—kefirs; SC—sour cream.

A multivariate statistical procedure was applied to determine the similarity in COP contents and profiles among the examined dairy products. The results are presented in Figure 3. Cluster analysis (CA) of oxysterol contents in the tested products revealed three clusters. The amount of all COPs combined caused the distinction in clusters (C1–C3; α = 0.05). The first cluster included mold cheeses and hard and semi-hard rennet cheeses (IM, EM, E, S, D, I). In the second cluster, processed cheese, sour cream and cottage cheese were placed. The last cluster consisted of yoghurt and kefir samples. Products included in the first cluster were characterized by high contents of 7K and 5,6βE, mean contents of 5,6αE and 7αOH, and a small content of 7βOH. Dairy products included in C2 typically had a high content of 7K, which was, however, smaller than in the case of C1, along with mean contents of both anomers, 5,6αE and 5,6βE, and low contents of 7αOH and 7βOH. Cluster C3 constituted fermented dairy products characterized by small contents of all determined COPs, among which the contents of 7αOH and 7βOH were the smallest.

Following a similar analysis of the COP profile, two clusters were identified (α = 0.05). The first cluster contained most cheeses and sour cream, and the second one included only yoghurt, kefir and processed cheeses.

## 4. Discussion

Dairy products are an important part of the daily diet for a large proportion of the population [22]. They are appreciated mainly for their specific taste and flavor [23]. A wide range of dairy products is available on the market to meet consumer needs. However, dairy products are a rich source of cholesterol and its oxidation products, which can be considered their natural contaminants [14].

In raw milk, the level of oxidized cholesterol derivatives is very low, sometimes undetectable by the commonly applied methods [24,25]. COPs are formed during technological processes [26], especially during fermentation/maturation, which may last for several years in the case of some types of cheeses. Therefore, despite the protective role of milk proteins and relatively high content of saturated fatty acids, which are not conducive to oxidation processes, a significant content of COPs can be expected in these products, which was confirmed in the present study [17]. Five oxysterols were found in the majority of the tested products, of which 7-ketocholesterol was dominant.

There are very few papers of other authors that present the contents and profile of oxidized cholesterol derivatives in milk and dairy products. Moreover, most of the available studies were published many years ago when less sensitive analytical methods were available and only one or two oxysterols could be determined [27].

In the work of Derewiaka and Obiedziński [28], oxysterol contents in selected fruity yoghurts were determined. The contents of 7K, 5,6βE and 7βOH detected in their study were similar to those in the present research. However, the authors detected only three oxidized cholesterol derivatives, which meant that the total COP contents they determined in yoghurts (13–18 mg kg^−1^ of fat) were lower than those detected in the present study. Similarly, in Schmarr, Gross and Shibamoto’s [29], work the total content of COPs in Parmesan amounted to 9 mg per kg of fat, whereas, in the present study, it reached 27 mg. The authors also found 19-hydroxycholesterol, 20α-hydroxycholesterol and 25-hydroxycholesterol in Italian-type cheeses. Nielsen, Olsen, Duedahl and Skibsted [30] determined the contents of oxysterols in dairy products by applying the HPLC technique. In the tested samples, five oxysterols were identified, similarly to in the present study. The contents of 7K, 5,6βE and 7βOH in yellow cheeses were similar to the contents noted in this paper (D, S, I, E), but the contents of epoxy cholesterol derivatives were lower. The authors also showed that the contents of oxysterols increase during the storage of cheeses.

Sander, Addis, Park and Smith [31] determined the contents of oxysterols in i.a. dairy products, but freeze-dried. In Cheddar cheese (2 samples out of 11) and parmesan (1 sample out of 6), they detected cholestanetriol and 25-hydroxycholesterol, which were not identified in any of the examined products in the current study. In one sample of sour cream, 5,6αE was the dominant oxysterol. In fresh cheese and sour cream, 7K was not detected. According to the results presented in this work, 7K is the dominant COP.

The data of previous studies are inconclusive. The differences among the results obtained by various authors may result from the different procedures of sample preparation or diversified analytical techniques. Till now, there has been no reference method for COP determination [18]. It should be noted that the analytical procedure of COP and cholesterol determination applied in the present study is fully validated and characterized by high sensitivity, repeatability and a wide linearity range for each tested compound [20]. Moreover, in the present paper, the total content of COPs, i.e., the sum of free and esterified compounds, was examined since saponification was one of the steps in the applied procedure. Instead, in some studies previously performed, only the free oxysterols present in a product were determined (completely omitting esterified forms of COPs) [17]; therefore, the total amounts of COPs determined were lower than those determined in the present research. This comprehensive approach to the analysis of COP content (both free and esterified) is emphasized as one of the main advantage of the present paper.

The results obtained in the present study indicated that the content of cholesterol and its oxidized forms was strongly dependent upon the amount of fat in the product. Hence, after re-calculating the COP content in order to express that in relation to fat (Figure 2), differences among product groups were demonstrated. These indicated that differences in technological processes, including the genus of microorganisms used, the length of maturation time as well as other external and internal factors, may affect the level of COPs in dairy products. It seems of utmost importance to identify as many factors as possible that determine the level of oxysterols in dairy products and to assess their exact impact as well as methods diminishing their formation and content in final products. Conscious management of these factors will make it possible to develop dairy products with reduced levels of cholesterol derivatives in the future. The benefit of such attempts will be to diminish the detrimental influence of COPs on health.

Particular attention should be paid to two product groups: yoghurt and kefir. These products were characterized by the lowest COP contents, which were associated with them having the lowest fat contents, and they had a different profile of COPs compared to cheeses. The total COP contents in these products were several times (even 24 times) lower than those in cheeses. Their daily consumption brings many benefits to the body, not only because of the contents of valuable nutrients but also because of the presence of probiotic microorganisms. However, the contents of COPs in cheeses are different, especially in cheeses with internal mold and Cheddar. The levels of these compounds are quite high. In addition, cheeses contain a lot of fat, in which saturated fatty acids predominate [32]. In addition, COPs from cheese can present a significant source of these contaminants in the diet. Previous studies have shown that dietary COPs can adversely affect human/animal bodies and cause a range of diseases, particularly inflammatory diseases such as atherosclerosis, neurodegenerative diseases and inflammatory bowel disease [33]. The pleiotropic effect of oxysterols on the human body results from the different properties of individual compounds [34]. The toxicities of individual COP are also not the same [35]. That is why it is important not only to determine the total number of COPs (or only one COP) as an indicator of cholesterol oxidation (such as 7K) but also to examine qualitatively and quantitatively all the cholesterol derivatives present in food.

The ratio of oxidized cholesterol derivatives to the parent compound determined in the present study was about 1.7%, which was slightly higher than those described in previously published papers. The majority of authors indicated the COP-to-cholesterol ratio to be around 1%, but it should be noted that there were dairy products available on the market for which this ratio could have been significantly higher [24,36]. However, as mentioned before, the evaluation procedure of the total content of COPs may be affected by different factors, which influence the final result.

The usage of a fully validated procedure confirms the quality and robustness of the obtained results and ensures that the correct inferences were drawn. The current study expands our knowledge about oxidized cholesterol derivatives in food of animal origin; however, further studies are needed regarding the intake of these compounds from the diet and their impact on health.

## 5. Conclusions

Dairy products are an important part of the diet for a large share of the general population. However, little is known regarding the contents of oxidized cholesterol derivatives in food, especially in dairy products. As a consequence, it is very difficult to estimate the daily dietary intake of these compounds. Unfortunately, their high consumption can contribute to an increase in the pool of these compounds in the human body, which may have severe health implications. Our results indicate, that fermented dairy products differ in contents of COPs, however they correlate with the total fat and total cholesterol content. Mold cheeses and hard and semi-hard rennet cheeses are the richest sources of COPs. Further research is needed to identify and characterize the external and internal factors that influence COP formation in dairy products, which is expected to contribute to a reduction in the levels of these contaminants in nutritionally valuable foods such as dairy products.

## Figures and Tables

**Table 1:** Research material: groups of cow’s dairy products that were tested.

Group Name	Abbreviation	Number of Samples
Cheeses with exterior mold (Camembert, Brie)	EM	12
Cheeses with internal mold (Blue, Gorgonzola)	IM	12
Dutch-type semi-hard cheeses (Gouda, Edam)	D	12
Swiss-type semi-hard cheeses (Emmental)	S	12
Italian-type very hard cheeses (Parmesan)	I	12
English-type hard cheeses (Cheddar)	E	12
Processed cheese	P	12
Curd cheeses/cottage cheese	C	12
Yoghurts	Y	12
Kefirs	K	12
Sour cream	SC	12

**Table 2:** Fat, cholesterol and cholesterol oxidation derivative (COP) contents in dairy products.

(*n* = 12 per Group)	$\bar{x}$ (SD)	EM	IM	D	S	I	E	P	C	Y	K	SC
Fat	[%]	27.5 ^b^	30.2 ^a,b^	26.8 ^b^	26.8 ^b^	28.8 ^b^	33.5 ^a^	18.8 ^c^	9.4	3.5 ^d^	1.8 ^d^	18.7 ^c^
		(4.4)	(3.9)	(1.4)	(1.0)	(1.5)	(1.2)	(6.5)	(2.2)	(1.8)	(0.3)	(1.6)
CH	[mg kg^−1^]	579 ^a,b^	639 ^a^	506 ^b,c^	499 ^c^	623 ^a^	625 ^a^	350 ^d^	308 ^d^	64 ^e^	38.4 ^e^	329 ^d^
		(42)	(99)	(77)	(48)	(75)	(61)	(64)	(61)	(34)	(9.1)	(49)
7K		3.03 ^a,b^	5.12 ^A^	2.52 ^b,c,A^	3.18 ^a,b,A^	2.58 ^b,c,A^	3.72 ^a,A^	2.57 ^b,c,A^	1.70 ^c,d,A^	0.35 ^e,A^	0.21 ^e,A^	1.54 ^d,A^
		(0.34)	(1.13)	(0.78)	(1.08)	(0.32)	(1.19)	(0.46)	(0.27)	(0.13)	(0.04)	(0.16)
7αOH		1.35 ^a,A^	1.40 ^a,B,C^	1.10 ^a–c,B,C^	1.16 ^a–c,C^	0.99 ^a–c,B,C^	1.29 ^a,b,B,C^	0.64 ^a–d,B^	0.39 ^c,d,B^	0.16 ^d,B^	0.08 ^d,B^	0.51 ^b–d,B^
		(0.16)	(1.16) *	(0.79) *	(0.75) *	(0.35) *	(0.80) *	(0.57) *	(0.41) *	(0.06)	(0.03) *	(0.32) *
7βOH		0.53 ^d,e^	0.97 ^a,C^	0.52 ^d,e,C^	0.73 ^b,c,C^	0.61 ^c,d,C^	0.88 ^a,b,C^	0.53 ^d,e,B^	0.39 ^e,B^	0.09 ^f,B^	0.07 ^f,B^	0.39 ^e,B^
		(0.05)	(0.22)	(0.07)	(0.18)	(0.05)	(0.26)	(0.21)	(0.05)	(0.03)	(0.03)	(0.06)
5,6αE		1.61 ^a–c,A^	2.19 ^a,B^	1.43 ^b–d,B^	2.10 ^a,b,B^	1.24 ^c–e,B^	2.18 ^a,B^	0.67 ^e,B^	1.08 ^c–e^	nd	nd	0.84 ^d,e^
		(0.78) *	(0.63)	(0.16)	(0.66)	(0.80) *	(0.41)	(0.73) *	(0.13)	nd	nd	(0.40) *
5,6βE		2.42 ^b^	4.15 ^a,A^	2.07 ^b,c,A^	2.71 ^b,A,B^	2.27 ^b,c,A^	3.58 ^a,A^	2.16 ^b,c,A^	1.50 ^c,A^	0.35 ^d,A^	0.21 ^d,A^	1.42 ^c,A^
		(0.26)	(0.91)	(0.15)	(0.90)	(0.28)	(1.61)	(0.40)	(0.15)	(0.12)	(0.07) *	(0.19)
∑ COPs		8.94 ^c,d^	13.82 ^a^	7.65 ^c,d^	9.88 ^b,c^	7.68 ^c,d^	11.65 ^a,b^	6.56 ^d,e^	5.06 ^e^	0.94 ^f^	0.57 ^f^	4.70 ^e^
		(1.43)	(2.46)	(1.66)	(3.34)	(1.28)	(3.48)	(1.36)	(0.82)	(0.30)	(0.11)	(0.50)
COPs/CH	[%]	1.54 ^b,c^	2.26 ^a^	1.51 ^b,c^	2.02 ^a,b^	1.26 ^c^	1.88 ^a,b^	1.93 ^a,b^	1.66 ^a–c^	1.38 ^c^	1.55 ^b,c^	1.46 ^b,c^
		(0.22)	(0.71)	(0.22)	(0.78)	(0.29)	(0.58)	(0.52)	(0.17)	(0.57)	(0.36)	(0.31)

CH—cholesterol; 7K—7-ketocholesterol; 7αOH—7α-hydroxycholesterol; 7βOH—7β-hydroxycholesterol; 5,6βE—5,6β-epoxycholesterol; 5,6αE—5,6α-epoxycholesterol/EM—cheeses with exterior mold; IM—cheeses with internal mold; D—Dutch-type cheeses; S—Swiss-type cheeses; I—Italian-type cheeses; E—English-type cheeses; P—processed cheese; C—curd cheeses; Y—yoghurts; K—kefirs; SC—sour cream. ^a–f^ homogeneous groups in rows; comparison among product groups (Tukey’s test, α = 0.05). ^A–C^ homogeneous groups in columns; comparison among COP contents in a specific product group (Tukey’s test, α = 0.05). The absence of a letter symbol means that the result is significantly different from the others. * not detected in all products in the group/nd—not detected.

**Table 3:** Correlation coefficients (r) between fat and cholesterol contents and the level of cholesterol oxidation derivatives (COPs) in dairy products.

	7K	7αOH	7βOH	5,6αE	5,6βE	∑ COPs
Fat	0.779	0.548	0.810	0.723	0.777	0.810
CH	0.768	0.594	0.786	0.735	0.749	0.807

CH—cholesterol; 7K—7-ketocholesterol; 7αOH—7α-hydroxycholesterol; 7βOH—7β-hydroxycholesterol; 5,6αE—5,6α-epoxycholesterol; 5,6βE—5,6β-epoxycholesterol.

## Data Availability

Dataset available on request from the authors.
